# Spatial multi-criteria decision analysis for the selection of sentinel regions in tick-borne disease surveillance

**DOI:** 10.1186/s12889-024-17684-x

**Published:** 2024-01-25

**Authors:** C. Guillot, C. Aenishaenslin, E. S. Acheson, J. Koffi, C. Bouchard, P. A. Leighton

**Affiliations:** 1 Groupe de recherche en épidémiologie des zoonoses et santé publique (GREZOSP), Department of Pathology and Microbiology, Faculty of Veterinary Medicine, University of Montreal, Montreal, Quebec, Canada; 2 Faculty of Medicine and Health Sciences, University of Sherbrooke, Sherbrooke, Quebec, Canada; 3 Centre de recherche en santé publique (CRESP) de l’Université de Montréal et du CIUSSS du Centre-Sud-de-l’Île-de-Montréal, University of Montreal, Montreal, Quebec, Canada; 4 Public Health Risk Sciences Divisions, National Microbiology Laboratory, Public Health Agency of Canada, Saint-Hyacinthe, Quebec, Canada; 5 Policy Integration and Zoonoses Division, Centre for Food-borne, Environmental and Zoonotic Infectious Diseases, Public Health Agency of Canada, Saint-Hyacinthe, Quebec, Canada

**Keywords:** Sentinel surveillance, Lyme disease, Lyme borreliosis, Multi-criteria decision analysis, Vector-borne diseases, Public health decision-making

## Abstract

**Background:**

The implementation of cost-effective surveillance systems is essential for tracking the emerging risk of tick-borne diseases. In Canada, where Lyme disease is a growing public health concern, a national sentinel surveillance network was designed to follow the epidemiological portrait of this tick-borne disease across the country. The surveillance network consists of sentinel regions, with active drag sampling carried out annually in all regions to assess the density of *Ixodes* spp. ticks and prevalence of various tick-borne pathogens in the tick population. The aim of the present study was to prioritize sentinel regions by integrating different spatial criteria relevant to the surveillance goals.

**Methods:**

We used spatially-explicit multi-criteria decision analyses (MCDA) to map priority areas for surveillance across Canada, and to evaluate different scenarios using sensitivity analyses. Results were shared with stakeholders to support their decision making for the selection of priority areas to survey during active surveillance activities.

**Results:**

Weights attributed to criteria by decision-makers were overall consistent. Sensitivity analyses showed that the population criterion had the most impact on rankings. Thirty-seven sentinel regions were identified across Canada using this systematic and transparent approach.

**Conclusion:**

This novel application of spatial MCDA to surveillance network design favors inclusivity of nationwide partners. We propose that such an approach can support the standardized planning of spatial design of sentinel surveillance not only for vector-borne disease BDs, but more broadly for infectious disease surveillance where spatial design is an important component.

**Supplementary Information:**

The online version contains supplementary material available at 10.1186/s12889-024-17684-x.

## Background

Tick-borne diseases (TBDs) represent a major concern for public health globally. The geographical expansion of tick populations has resulted in increased incidence of diseases such as Lyme disease (LD), anaplasmosis, and tick-borne flaviviruses (e.g., tick-borne encephalitis), to name a few [1–4]. TBDs can affect humans, domestic animals, and wildlife, leading to far-reaching impacts on our societies [4]. Amongst TBDs, Lyme disease (LD) is the most common vector-borne disease found in the northern hemisphere [5–8].

LD is caused by a variety of genospecies of *Borrelia burgdorferi* senso lato [9]. Tick vectors of LD belong to the *Ixodes* genus but differ in species according to geographical location. In North America, the principal vectors are *I. scapularis* and *I. pacificus,* in Europe they are *I. ricinus* and *I. persulcatus,* and in Asia, the main tick species of interest is *I. persulcatus* [9, 10]. The spatial spread of ticks by host animals (e.g., deer, birds), leading to the local establishment of new tick populations, is a key mechanism driving the geographic expansion of LD risk [11, 12]. Thus, the surveillance of ticks is used to monitor the increase in *Borrelia* spp. and other pathogens carried by blacklegged ticks in human and animal populations [13, 14].

Acarological active surveillance can be used to detect the presence of tick populations in the environment [15, 16]. This method usually consists of drag or flag sampling in ecologically suitable sites (i.e., consisting of deciduous or mixed forests). The density of infected nymphs questing in the environment can be calculated, and this measure has been correlated with LD risk to human populations [14, 17]. However, due to the intensive nature of active surveillance, the surveillance zone must be carefully targeted [7, 18]. In Europe, several surveillance scenarios were assessed by Eurosurveillance to give insight into which methods would lead to more effective and efficient surveillance [19]. In this review, active surveillance of ticks was deemed a complicated process, with difficulties involving timely, standardized sampling across a substantial study area [19]. Large-scale standardized acarological active surveillance systems for LD (e.g., at the national or continental scales) are, to our knowledge, yet to be developed due to important feasibility issues, although extensive tick surveillance systems have been put in place (e.g., in the United States). However, such systems have the potential to provide a comparable measure of acarological hazard across space and give insight into the evolving portrait of tick population establishment and TBD risk emergence.

In Canada, human LD cases have been increasing exponentially in the last decade. In 2010, 143 cases were diagnosed and, by 2021, this number reached nearly 3000 [20]. In parallel, *Ixodes* spp. tick populations have expanded their geographical range within Canada [16, 21, 22]. As a result of this range expansion, the federal passive surveillance system initiated in the early 1990s experienced an increasing volume of tick submissions, which overwhelmed the national and provincial public health laboratories. Therefore, this passive surveillance system was discontinued in 2021. Thus, to survey the acarological risk of LD in Canada, active surveillance efforts now represent the main source of validated information. Currently, active surveillance efforts in Canada are coordinated at the provincial or regional level and performed by public health authorities or academia; therefore, funding, protocols, and surveillance efforts vary greatly across the country. The expansion of the geographic range of infected blacklegged ticks with various pathogens and the risk that it poses for the health of the Canadian population highlights the need for developing a national level active surveillance network. Such a surveillance network should be able to track acarological hazards (i.e., changes in abundance of ticks) in space and time to alert public health authorities and can indicate the need for public health interventions. Furthermore, active surveillance permits PCR testing and could indicate novel circulation of an emerging pathogen.

With the increasing public concern related to LD, the development of a Canadian Lyme Sentinel Network (CaLSeN) was proposed by the Canadian Lyme Disease Research Network (CLyDRN) as part of its ‘Prevention and Risk Reduction’ pillar. The objective of the network is to follow the epidemiological portrait of LD across the 10 Canadian provinces, using active surveillance (drag sampling) to measure tick density and assess the occurrence of *B. burgdorferi* as well as other tick-borne pathogens in the environment. However, due to the vastness of the defined surveillance zone, active surveillance of this large territory represents a logistical challenge.

Canada has a surface area of nearly 10 million km^2^, making it the second-largest country in the world by area, with a population of over 35 million [23]. To survey large geographical areas using active surveillance, a sentinel approach can make the endeavour feasible. Sentinels are a fixed subset of units selected from the defined source population, sampled repeatedly through time to follow spatial and/or temporal disease trends. In the context of acarological active surveillance, sentinels take the form of sentinel sites; these sites are visited regularly so that tick densities are monitored spatio-temporally. A vast range of considerations fuel the reflection on how to distribute sentinels across the study zone, including known presence of risk, environmental suitability, and logistical constraints [24]. However, during the decision-making step, the retained criteria are unlikely to all be of equal importance, a problem that needs be taken into consideration during the planning phases of the surveillance system.

Multi-criteria decision analysis (MCDA) approaches provide a systematic and objective strategy to deal with such a dilemma. MCDA is used in several fields, including economics, politics, and health, to support decision making in complex situations involving multiple and even conflicting objectives [25]. MCDA has been used in the past for comparison of management plans for TBDs, including communication, surveillance, and control strategies [26–29]. Results highlighted the ability of MCDA to characterize the key issues and complexities regarding TBDs and include them in decision making. As an extension to classic MCDA, the incorporation of georeferenced data via Geographic Information Systems (GIS) can provide a spatial representation of the prioritization process emanating from the analysis. Such a strategy (GIS-MCDA) has been proposed for the public health management of vector-borne diseases in general and specifically for TBD surveillance [30, 31].

In this article, we apply a GIS-MCDA to prioritize surveillance regions across Canada for the spatial design of a new national sentinel surveillance system for ticks and TBDs (CaLSeN). This study aims firstly, to use GIS-MCDA to prioritize sentinel regions by integrating different spatial criteria relevant to the surveillance goals; and secondly, to use the resulting prioritization map to inform decision-making to support them in selecting areas suitable for active tick surveillance. This method could be adapted to meet surveillance needs for other vector-borne diseases, or even other infectious diseases, in other geographical areas.

## Methods

### The Canadian Lyme sentinel network (CaLSeN)

CaLSeN uses a standardized protocol to map reported LD cases across Canada. Within the network, the surveillance units are “sentinel regions”. Sentinel regions are circular areas with a radius of 50 km around a population center. Each node is composed of 5 to 10 sampling sites, which are visited yearly to drag for ticks and collect ecological data. CaLSeN was first piloted in the summer of 2019 to assess the feasibility of sampling across Canada [32]. Sampling did not go ahead in 2020 due to travel restrictions in place with response to the COVID-19 pandemic. The network was subsequently expanded for 2021 and 2022, using a spatial MCDA approach to support sentinel region selection.

### Spatial MCDA process

**Fig. 1:**
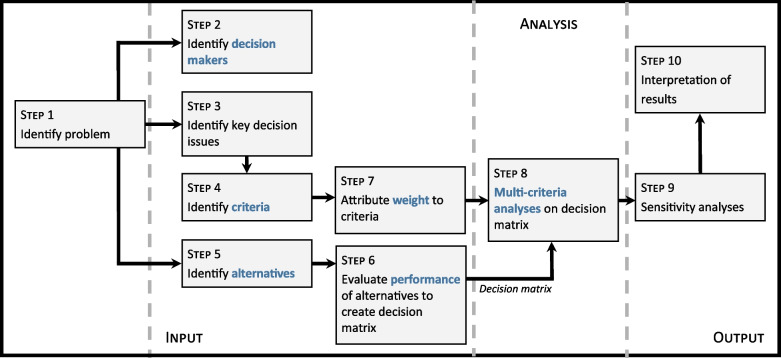
Diagram of general steps in multi-criteria analysis

The MCDA process can be divided into 10 systematic steps (Fig. 1). It requires three main elements, or inputs, which must be defined: the decision makers, the criteria, and the alternatives [33]. Several key concepts underpin the analysis: weighting, performance evaluation, and combination rules (or aggregation). The spatial extension to GIS-MCDA translates the data into georeferenced layers within a geographic information system to provide a spatially-explicit solution to the problem.

#### Step 1. Identify the problem

First, the problem must be clearly identified to allow stakeholders to work towards a similar goal. This step allows project leads to identify relevant decision makers, criteria, and alternatives (see Steps 2 to 5). For CaLSeN, the problem was the need to identify relevant and feasible sentinel regions for the active surveillance of ticks across Canada, including presence and abundance of ticks and pathogen prevalence. Thus, it was determined that a spatial MCDA would use spatially explicit data and the output of the analysis would support decision making through the production of maps.

#### Step 2. Identify decision makers

Decision makers (DMs) were identified based on their participation in ongoing tick-borne disease research, their active role in CLyDRN and their expertise in tick and LD surveillance. A total of 13 DMs were identified and agreed to participate in the study. Each province was represented by at least one DM. The panel of professionals was composed of academics and provincial and federal public health authorities.

#### Steps 3–4. Identify decision issues and criteria

A decision tool had been previously developed to help researchers and public health authorities decide on geographical positioning of sentinel locations for vector-borne disease- surveillance [34]. The output of the tool is a list of criteria which should be considered during site selection. The tool was applied to our case study and performance measures were developed from the retained criteria (Table 1). Each criterion was translated into a vector layer in QGIS version 3.18.1 [35] using available georeferenced data (see Step 7).

**Table 1:** Criteria used in the MCDA for selection of sentinel regions of the Canadian Lyme Sentinel Network (CaLSeN) with performance measures

No.	MCDA criteria	Performance measure
1)	Maximize the human population reached within the units of the study zone	Logarithm of the population taken from Statistics Canada’s Census 2016 data
2)	Document risk of disease due to the presence of appropriate vector within the sentinel region	Number of passive tick submissions from federal passive surveillance system from 2010 to 2015 standardized by the logarithm of the population
3)	Determine ecological suitability for the presence of the vector, *Ixodes* spp. ticks	Habitat suitability indication for *Ixodes* spp. ticks using the product of the percentage of deciduous or mixed forest cover and temperature in the form of accumulated degree days above 0 °C
4)	Identify logistical constraints	Distance traveled between the nearest CLyDRN collaboration center (i.e. main address of sampling teams) to the center of the sentinel region in kilometers

#### Step 5. Identify alternatives

Alternatives are all potential solutions to the decision problem. As stated in Steps 3–4, for each of the criteria used for the MCDA process, georeferenced data were used to measure the performance of each criterion. The use of georeferenced data layers permitted the use of all of southern Canada as an alternative for the identification of sentinel regions, without being impeded by administrative boundaries. Thus, the alternatives in this case study are any point in space in southern Canada, which could then be considered as a potential site for a sentinel region. Southern Canada represents the area at a maximum of 600 km from the United States border, where the risk of LD is present or emerging. The alternatives covered by the GIS-MCDA were also restricted to permit better visual differentiation between rankings in the high-risk area.

The vector layer for each criterion was converted to a raster layer with a cell size of 25 km in QGIS, a size that was considered to strike a suitable balance between specificity and sensitivity for criteria performance; for point data (e.g., passive surveillance and human population data), a cell size that is too small would lack sensitivity for evaluating the performance of the criterion, whereas a cell size that is too large would result in lower specificity. The rasters contained a performance value for each cell size and the georeferenced cells were thus used as the geographical unit of the alternatives.

#### Step 6. Attribute weights

Each of the DMs was asked to weigh the criteria by allocating 100 points between the four criteria. Hence, each DM would allocate a higher score to the criterion they considered most crucial for decision-making and a lower score to criteria they deemed less important, keeping in mind that they cannot award more than 100 points. Individual results were kept hidden from the group to ensure DMs were not influenced by each other. Final weights were presented to DMs during the consultation process to gain a consensus on final weights which would be applied to the MCDA. DMs decided that the final weight values for each criterion would remain the same for each model to support a standardized approach to sentinel unit selection.

#### Step 7. Evaluate performance

A performance score was attributed for each alternative for each of the criteria, as described in subsequent paragraphs. The performance score is the value of each criterion for each alternative. For example, for the number of inhabitants, one city may have a score of 1000 if it has 1000 inhabitants, while another city will have a score of 500 if it has 500 inhabitants. The combination of the performance scores for the criteria for each of the alternatives is known as the decision matrix.

For the first criterion, human population data were obtained from Statistics Canada’s 2016 Census [23]. Polygons were created using publicly available census subdivision (CSD) boundaries from Statistics Canada [23], with population data georeferenced to the centroid of the polygon.

The second criterion was addressed using passive acarological surveillance data made available through data sharing agreements from each province. In Canada, passive surveillance data involves voluntary submissions of ticks found attached to a patient, by healthcare professionals, pharmacies, veterinarians or the patient themselves. The data are georeferenced to the CSD centroid of the municipality where the tick was acquired. Number of passive tick submissions originating from humans between 2005 and 2015 were used. For most of the provinces, a tick index was derived using these data [36] by summing the number of tick submissions from 2005 to 2015 and dividing by the logarithm of the population. However, this measure was deemed inappropriate for provinces where passive surveillance was discontinued in regions of high submissions. These included Ontario, Quebec, and Nova Scotia. For these provinces, a second establishment period index was developed. Koffi et al. (2012) identified a threshold of passive tick submissions associated with the presence of questing ticks in the environment during active surveillance within a given CSD. Leighton et al. (2012) then applied this threshold to identify CSDs with a high likelihood of containing an established tick population as those which exceeded the threshold for two consecutive years, since persistent observations of high tick submissions provided stronger biological evidence of a locally reproducing tick population. We applied the approach of Leighton et al. (2012), analyzing the passive surveillance dataset to identify years from 2000 to 2015 in which tick submissions from each CSD exceeded a threshold of one tick submission per logarithm of the population and cumulating “years of establishment” following the second consecutive year in which the threshold was exceeded [37]. This empirical cut-off was determined by evaluating the risk distribution across CSDs by province and selecting a threshold which was discriminatory, and which allowed within province comparisons (Supplementary material 1). The final index was thus a duration-of-establishment period, in years, which was used as a measure of risk for these provinces.

The third criterion, determining the ecology of the territory to allow for the establishment of ticks, was addressed using shapefile data of land cover across Canada. Ticks can establish in a range of habitats [38]; however, woodlands are generally considered most suitable for *Ixodes *spp. ticks [39]. To increase specificity of these criteria, we decided to include data on mixed or deciduous forest as these forest types are particularly associated with the presence of *Ixodes* spp. ticks [40]. Land cover data from 2015 [41] were used to calculate the percentage of forests for each 25 km grid squares across the study zone. Annual accumulated degree days (DD) > 0 °C were calculated for each 25 km grid square using climate normal data (1981–2010 averages) from ClimateNA [42], and the product of percentage of forest with DD > 0 °C was used as a habitat suitability index for tick populations [43].

The fourth criterion considers the logistics of sampling in the form of the proximity between the potential sentinel regions and a collaboration center. The location of the CLyDRN center was added to the map and a distance matrix in kilometers was created between these collaboration centers and potential sentinel regions. For Newfoundland and Labrador and Prince Edward Island, partners available to carry out sampling activities were not identified at the time of the decision-making process. For these provinces, this last criterion was thus omitted.

According to the MCDA algorithm chosen, the decision matrix should be normalized [44]. Thus, for each province, each of the criteria were standardized by mean and standard deviation according to Formula 1:

1 $$z=\frac{X-\mu }{\sigma }$$

where z is the standardized number, x is the raw figure, μ is the group mean, and σ is the group standard deviation.

#### Step 8. Apply combination rules

Combination rules refer to the way the algorithm runs mathematically and is also referred to as aggregation. There are many different MCDA algorithms, and it has been shown that methods vary greatly between studies [45–47]. Thus, frameworks have been developed to guide decision makers on which method they should use based on their research objectives and the level of uncertainty in their data [48]. According to the framework developed by Wątróbski et al.*,* [48], the PROMETHEE II method, using pairwise comparisons, should be used in our case study based on the decision problem descriptors.

The visual PROMETHEE Academic Edition [49, 50] was used to run the models. Ten models were created: one for each province. This enabled a comparison of the MCDA results at the provincial level, facilitating the identification of sentinel regions within that specific province. PROMETHEE II complete rankings were chosen, and as the model does not allow for incomparabilities, all alternatives were ranked by the algorithm. Complete rankings were deemed appropriate as there were no strongly conflicting criteria [50]. The chosen output of the analysis for the models was the global Phi score, where the highest Phi represents a better scoring alternative in the MCDA.

#### Step 9. Carry out sensitivity analyses

The final step prior to interpretation of results is the sensitivity analysis. The visual stability intervals function in Visual PROMETHEE indicates the range in which the weight of a criterion can be modified without affecting the ranking for a given scenario. This allows the evaluation of the robustness of the prioritization based on the weighting of the criteria. Furthermore, three alternative scenarios were created to provide a visual cartographic representation of the impact of each of the criteria including risk-based, environmental, and population scenarios (Table 2).

**Table 2:** Weights attributed to the three alternative scenarios used for sensitivity analyses

Scenario	Weights (%)			
	Risk	Environment	Population	Distance
A) Risk-based	70	10	10	10
B) Environmental	10	70	10	10
C) Population	10	10	70	10

#### Step 10. Interpret results

Lastly, the Phi scores were imported into QGIS version 3.18.1 to create maps to represent the highest Phi score using the analyses. The SAGA Gaussian filter was used to smooth grid data and remove noise, where the degree of smoothing is dependent on standard deviation [51].

These maps were presented back to DMs during follow-up meetings for final decisions on sentinel region locations. Meetings were held in large groups, with all provinces attending, but also at the provincial level. Using the maps, population centers were identified which consisted of areas of key scientific interest for the establishment of a sentinel region. Sensitivity analyses were used to support decision making.

## Results

### Weighting

After individual criteria were weighted and an interactive session was held to obtain a consensus from all parties, final weights were calculated. These final weights are presented, along with the standard deviation, in Table 3.

**Table 3:** Final weights attributed to each of the criteria. Final weights were individually scored, then a mean was calculated, rounded to the nearest percentage, and presented back to the group to gain consensus on the final weights. Standard deviations are also shown to demonstrate the spread in weighting

Criteria	Weight	Standard deviation
Acarological risk	40	9.81
Log of the population	25	7.34
Environmental index	25	8.86
The distance from collaborating research centers	10	3.08

### Phi scores for MCDA

**Fig. 2:**
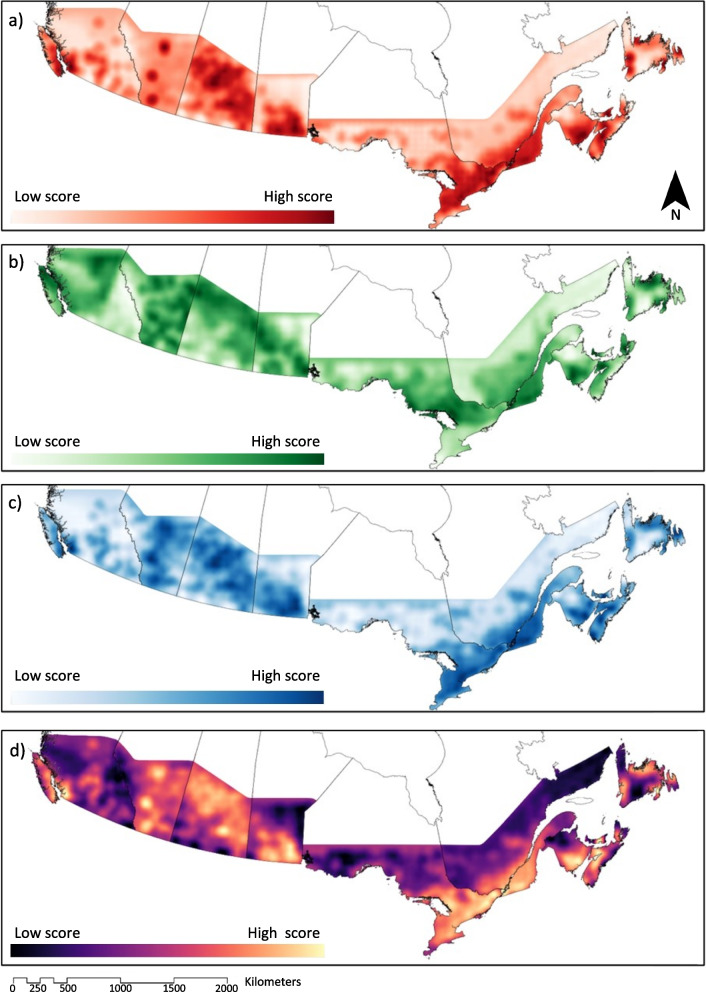
Maps projecting MCDA Phi scores from scenario **a**) risk-based, **b**) population, **c**) environment, and **d**) weighted. The shading indicates the relative performance across the set of alternatives – a higher score represents a better performance according to the criteria and weighting used within the models. Specifically, scores represent how performance is distributed across space at the provincial level according to the MCDA, depending on criteria weightings. These maps were presented back to stakeholders to support decision-making for selection of sentinel regions

MCDA were run for each of the provinces, and the Phi values from the varying scenarios were used to create maps (Fig. 2). These maps were presented to stakeholders to support decision making for the selection of sentinel regions.

### Sensitivity analyses

Sensitivity analyses were performed to assess the impact of weighting scores on the prioritization of sentinel regions by province (Supplementary material 2). As an example of the exercise, we present visual stability intervals using results generated from the Prince Edward Island data (Table 4). The stability intervals are presented for each of the four evaluation criteria and permit the evaluation of how rankings would be affected if the weight attributed to the criterion in question was altered.

**Table 4:** Stability levels for results of Prince Edward Island for weighted scenario, with levels for all ranking to remain the same, and levels for half of the rankings to remain the same

Criteria	Weight	Stability intervals^a^ for rankings to remain the same		Stability intervals for 50% of rankings to remain the same	
		Min weight	Max weight	Min weight	Max weight
**Risk**	40	29.4	42.9	29.4	46.3
**Environment**	25	17.8	34.8	17.8	34.8
**Population**	25	18.9	44.4	0	44.4
**Distance**	10	7.7	21.7	3.1	21.7

^a^Stability intervals refer to range in which the weight of a criterion can be modified without affecting the ranking for a given scenario

### Interpretation of results

**Fig. 3:**
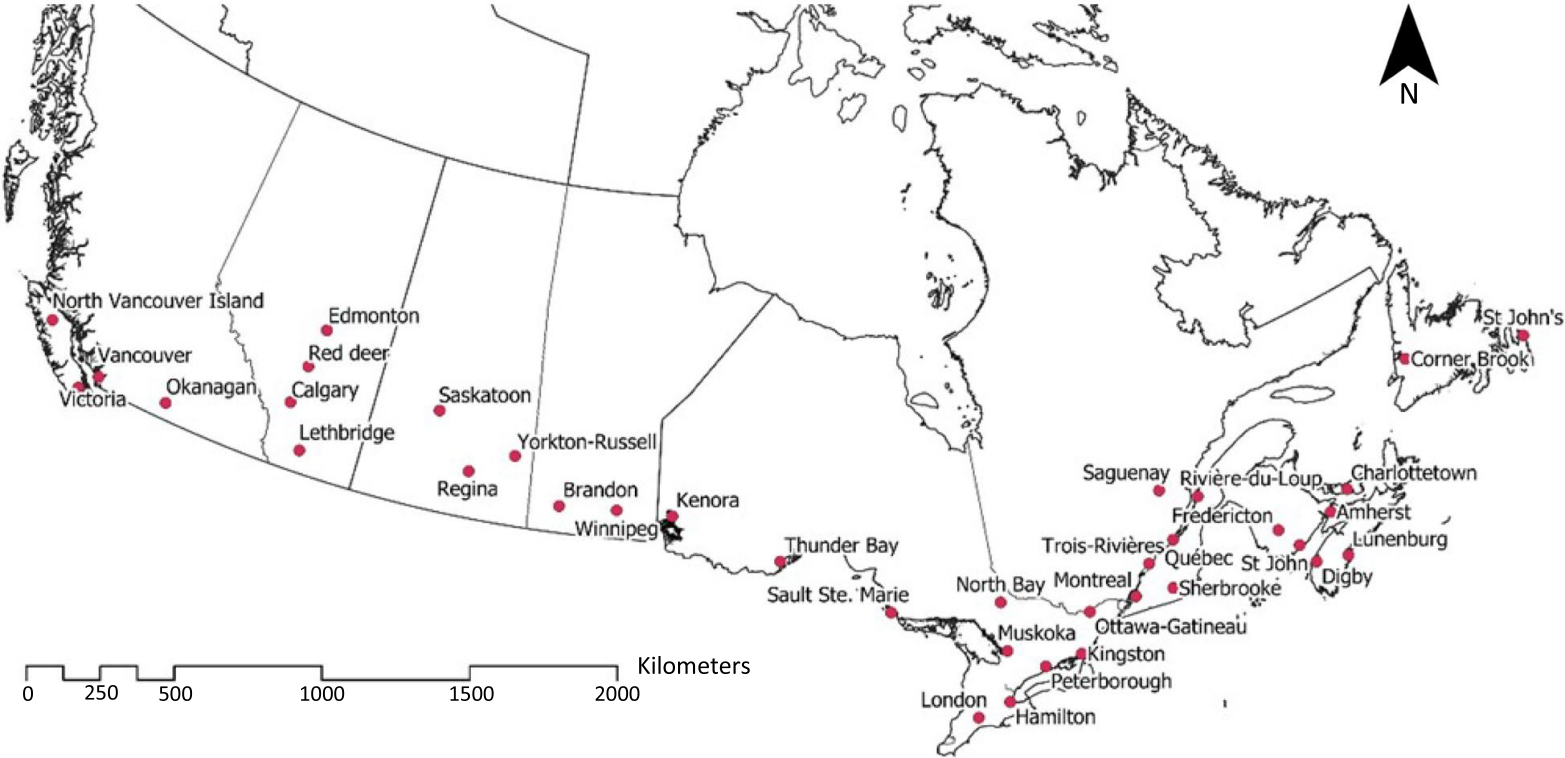
Sentinel regions (*n* = 37) for the Canadian Lyme Sentinel Network (CaLSeN) (pink dots)

Three meetings with all DMs were held, in addition to one or two meetings for each province. During these meetings, the maps were used as a decision support tool for selecting sentinel regions, focused around a population center. Following these multiple group discussions, a final map displaying which regions had been retained as part of the sentinel surveillance network (Fig. 3) was presented to the whole Surveillance Working Group. A final consensus was gained for the spatial distribution of sentinel nodes across Canada.

Following the sensitivity analyses, results were presented to the DM group to provide visual support for decision making. For each province, the number of desired sentinel regions was decided by the group according to the resources that each province could attribute to sampling.

## Discussion

With the growing burden of vector-borne diseases, accelerated by climate and anthropogenic changes, effective surveillance systems must be put in place to track the associated evolving risk [52]. We have employed a spatial MCDA approach to target 37 areas of scientific interest for active tick surveillance of LD risk throughout Canada and inform stakeholders. This novel approach makes it possible to take into consideration multiple facets related to the complex life cycles of TBDs, such as human population density, environmental suitability, and logistical constraints.

Emergence of vector-borne diseases is characterized by a complex epidemiological process, requiring an interaction between pathogens, vectors, susceptible animal hosts, and human populations [53]. With uneven distribution of suitable ecology for the vector and pathogen establishment process, and human populations centered around urban centers, the risk for vector-borne diseases is spatially heterogenous [52]. This creates significant challenges in constructing informative and representative surveillance systems, especially at a large scale, and requires an important decision-making effort during the planning stages. The MCDA allows for a systematic, inclusive, and transparent approach for selecting sentinel regions whilst considering relevant, but sometimes conflicting, criteria [25, 26, 33].

By creating different scenarios, we can understand the impact of each criterion on the final MCDA output using visual tools and use this information to inform DMs. This approach has permitted us to see which geographical areas incorporate the facets most related to Lyme disease surveillance priorities and use the maps as a tool for the decision process. Through the sensitivity analyses, we were able to determine the stability intervals for the choosen weightings of each of the sentinel regions. In this case study, it was seen that criteria weightings had a large impact on final rankings for several provinces (Supplementary material 2), highlighting the importance of reaching an agreement in the weights attributed to each of the criteria by DMs for consensual decision-making. Data quality can also have an important impact on final results of MCDA models. Although passive surveillance has been found to be a good indicator of LD risk to human populations [14], sources of error may be introduced when specifying the precise locations of tick encounter. Furthermore, changes in passive surveillance protocols across time has meant that our risk measures in several provinces have had to be adjusted, generating other sources of error. Conducting sensitivity analyses to understand the possible alternative outcomes can thus support decision-making by offering a broader perspective of the situation, as opposed to relying on a single input which is not immune to these sources of error. In the case of surveillance data, its potential use in complex decision-making highlights the need to continue to optimize and systematize the data generated from surveillance systems.

As the MCDA exercise has permitted our group of DMs to establish final sentinel regions, next steps will be to distribute sampling sites throughout each sentinel region. Previous sentinel networks have used grid separation to gain even geographical representation of the study area. These sites will serve as transects for drag sampling and allow for multiple data points to be collected across the sentinel regions to obtain finer scale acarological risk data. A standardized sampling protocol will then be applied at each of the sampling sites.

An important aspect of using a MCDA approach is that the output of the analyses is used as a decision aid support tool, as opposed to simply creating a final decision map [30]. This allows flexibility in the decision-making process and permits decision makers to reflect upon priorities and how to distribute sentinels across the study area to gain the best geographical representation whilst optimizing the relevance of the sentinel regions selected.

A limitation of using an MCDA approach in the decision-making process is the substantial effort required to recruit and involve a variety of experts. Although input from different decision makers represents a strength of the process, the coordination and numerous feedback loops of the process represent a significant investment of time for those involved. For the establishment of short-term surveillance networks, for instance for surveillance of outbreaks, this method may not be appropriate. However, in the context of establishing a long-term surveillance network, contribution from experts in the field assures that sentinel regions will be relevant for a long period of time.

## Conclusions

Our study showcases an innovative application of spatial MCDA for the establishment of a nationwide surveillance system. This has allowed us to pinpoint areas of key surveillance interest across the country in a flexible manner, as the LD emergence status is not equivalent across the country. The results of this exercise have been used to support complex decision-making regarding the establishment of a sustainable national surveillance program for LD in Canada. This approach could benefit other stakeholders, such as public health authorities or academics, during the elaboration of new vector-borne disease sentinel surveillance networks.

### Supplementary Information

**Additional file 1.** Supplementary Material 1.

**Additional file 2.** Supplementary Material 2.

## Data Availability

Data related to population density and forest cover across Canada are publicly accessibly via Statistics Canada and Land Cover Canada, respectively. The datasets for passive surveillance used in this study were not collected nor owned by the authors, and thus cannot be shared publicly. The datasets are held by the Public Health Agency and the British Columbia Center for Disease Control. These institutions should be contacted directly to request access to datasets.

## Abbreviations

CaLSeN: Canadian Lyme Sentinel Network
CLyDRN: Canadian Lyme Disease Research Network
CSD: Census subdivision
DMs: Decision-makers
LD: Lyme disease
MCDA: Multi-criteria decision analysis
PHAC: Public Health Agency of Canada
TBDs: Tick-borne diseases
