# Supplementary material for: Spatial multi-criteria decision analysis for the selection of sentinel regions in tick-borne disease surveillance
Source: BMC Public Health. 2024 Jan 25;24:294. doi: 10.1186/s12889-024-17684-x (PMC10809750; doi:10.1186/s12889-024-17684-x)
Supplement: Supplementary file 1 — Additional file 1. Supplementary Material 1. [file 12889_2024_17684_MOESM1_ESM.pdf]

Supplementary material: Threshold values for Lyme disease risk in provinces where passive surveillance was discontinued within some localities

The data used to represent risk of Lyme disease for the multi-criteria decision analyse was passive surveillance submissions (number of ticks submitted per logarithm of the population). For most of the provinces, a tick index was derived using these data (Koffi *et al.*, 2012) by summing the number of tick submissions over a period of ten years, from 2005 to 2015, divided by the logarithm of the population.

However, this measure was deemed inappropriate for provinces where passive surveillance was discontinued in regions of high submissions. These included Ontario, Quebec and Nova Scotia. For these provinces, a second establishment period index was developed. Koffi *et al.* (2012) identified a threshold of passive tick submissions associated with the presence of questing ticks in the environment during active surveillance within a given CSD. Leighton *et al.* (2012) then applied this threshold to identify CSDs with a high likelihood of containing an established tick population as those which exceeded the threshold for two consecutive years, since persistent observations of high tick submissions provided stronger biological evidence of a locally reproducing tick population.

We applied the approach of Leighton et al. (2012), analyzing the full passive surveillance data set to identify years from 2000-2015 in which tick submissions from each CSD exceeded a threshold of 1 tick submission per logarithm of the population and cumulating "years of establishment" following the second consecutive year in which the threshold was exceeded (Leighton *et al.,* 2012)). This empirical cut-off was determined by evaluating the risk distribution across CSDs by province and selecting a threshold which was discriminatory, and which allowed within province comparisons (Figure 1). The final index was thus a duration-of-establishment period, in years, which was used as a measure of risk for these provinces.

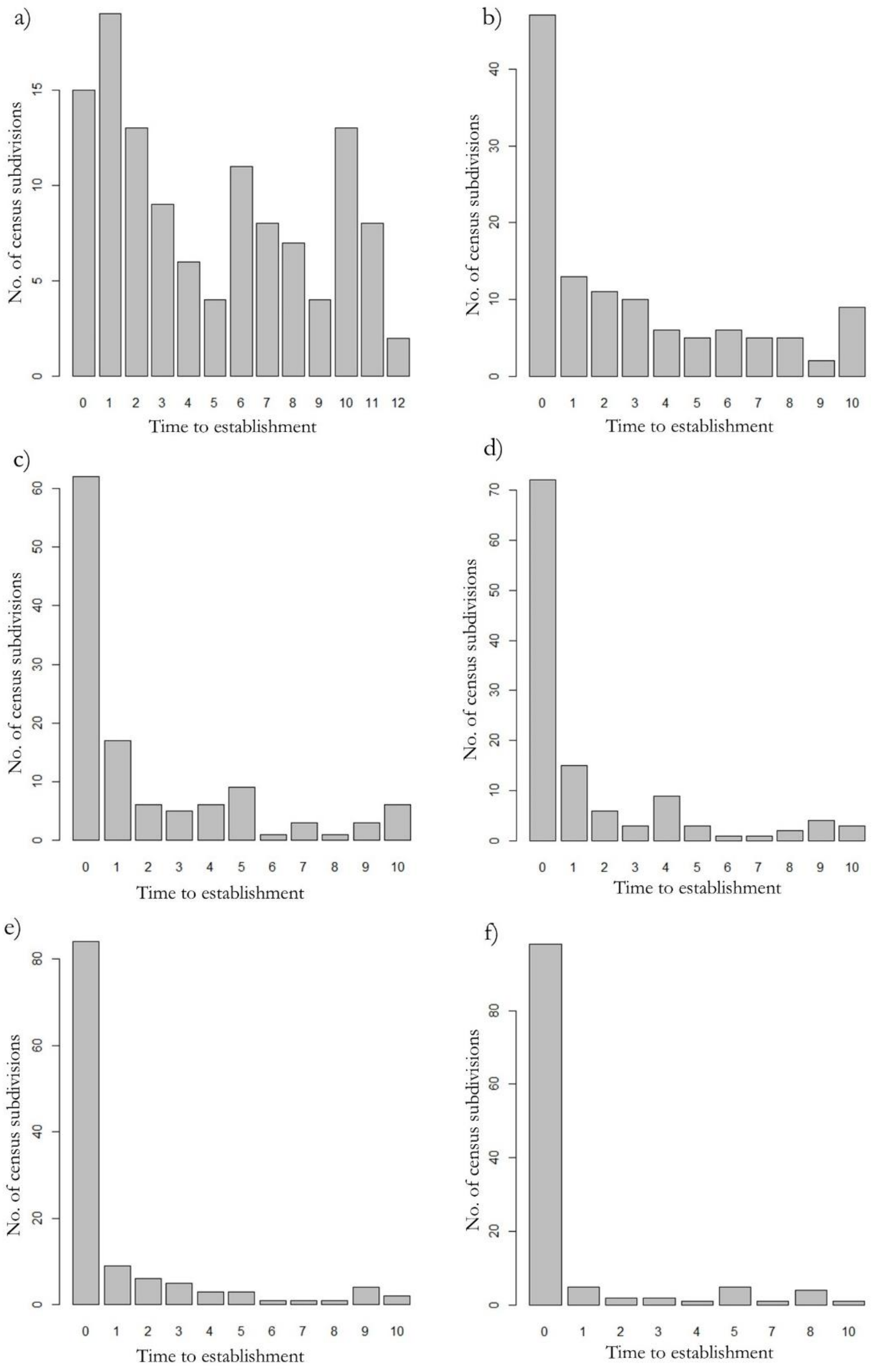

Figure 1. Distribution of Lyme disease risk, in the form of duration-of-establishment period of *Ixodes scapularis*, according to different thresholds a) 0.1 tick submission per logarithm of the population b) 0.5 tick submission per logarithm of the population c) 1 tick submission per logarithm of the population d) 1.5 tick submission per logarithm of the population e) 2.5 tick submissions per logarithm of the population f) 5 tick submissions per logarithm of the population

References

Koffi JK, Leighton PA, Pelcat Y, Trudel L, Lindsay LR, Milord F, et al. Passive surveillance for I. scapularis ticks: enhanced analysis for early detection of emerging Lyme disease risk. Journal of medical entomology. 2012;49(2):400-9

Leighton PA, Koffi JK, Pelcat Y, Lindsay LR, Ogden NH. Predicting the speed of tick invasion: an empirical model of range expansion for the Lyme disease vector Ixodes scapularis in Canada. 2012;49(2):457-64
