# Supplementary material for: Spatial multi-criteria decision analysis for the selection of sentinel regions in tick-borne disease surveillance
Source: BMC Public Health. 2024 Jan 25;24:294. doi: 10.1186/s12889-024-17684-x (PMC10809750; doi:10.1186/s12889-024-17684-x)
Supplement: Supplementary file 2 — Additional file 2. Supplementary Material 2. [file 12889_2024_17684_MOESM2_ESM.pdf]

Table 1. Stability levels for results of **British Columbia** for weighted scenario, with levels for all ranking to remain the same, and levels for half of the rankings to remain the same.

Criteria	Weight	**Stability intervals* for rankings to remain the same**		**Stability intervals for 50% of rankings to remain the same**	
		Min weight	Max weight	Min weight	Max weight
**Risk**	40	39.9	40.2	6.3	43.8
**Environment**	25	25.0	25.0	21.9	52.9
**Population**	25	25.0	25.0	12.8	47.9
**Distance**	10	10.0	10.0	4.6	29.7

* Stability intervals refer to range in which the weight of a criterion can be modified without affecting the ranking for a given scenario

Table 2. Stability levels for results of **Alberta** for weighted scenario, with levels for all ranking to remain the same, and levels for half of the rankings to remain the same.

Criteria	Weight	**Stability intervals for rankings to remain the same**		**Stability intervals for 50% of rankings to remain the same**	
		Min weight	Max weight	Min weight	Max weight
**Risk**	40	36.1	42.8	19.4	45.4
**Environment**	25	25.0	25.0	23.9	22.2
**Population**	25	25.0	25.0	23.1	26.3
**Distance**	10	10.0	10.0	4.3	11.8

Table 3. Stability levels for results of **Saskatchewan** for weighted scenario, with levels for all ranking to remain the same, and levels for half of the rankings to remain the same.

Criteria	Weight	**Stability intervals for rankings to remain the same**		**Stability intervals for 50% of rankings to remain the same**	
		Min weight	Max weight	Min weight	Max weight
**Risk**	40	39.1	41.0	15.7	41.0
**Environment**	25	25.0	25.0	23.4	27.6
**Population**	25	25.0	25.0	22.4	31.3
**Distance**	10	10.0	10.0	8.1	12.5

Table 4. Stability levels for results of **Manitoba** for weighted scenario, with levels for all ranking to remain the same, and levels for half of the rankings to remain the same.

Criteria	Weight	**Stability intervals for rankings to remain the same**		**Stability intervals for 50% of rankings to remain the same**	
		Min weight	Max weight	Min weight	Max weight
**Risk**	40	38.8	40.0	7.7	47.8
**Environment**	25	25.0	25.0	16.7	43.9
**Population**	25	25.0	25.0	0	40.64
**Distance**	10	10.0	10.0	2.6	11.1

Table 5. Stability levels for results of **Ontario** for weighted scenario, with levels for all ranking to remain the same, and levels for half of the rankings to remain the same.

Criteria	Weight	**Stability intervals for rankings to remain the same**		**Stability intervals for 50% of rankings to remain the same**	
		Min weight	Max weight	Min weight	Max weight
**Risk**	40	40.0	40.0	34.9	43.3
**Environment**	25	25.0	25.0	22.6	27.0
**Population**	25	25.0	25.1	21.7	30.5
**Distance**	10	10.0	10.0	3.1	11.8

Table 6. Stability levels for results of **Québec** for weighted scenario, with levels for all ranking to remain the same, and levels for half of the rankings to remain the same.

Criteria	Weight	**Stability intervals for rankings to remain the same**		**Stability intervals for 50% of rankings to remain the same**	
		Min weight	Max weight	Min weight	Max weight
**Risk**	40	40.0	40.0	28.6	57.1
**Environment**	25	25.0	25.0	25.0	38.8
**Population**	25	25.0	25.0	6.3	54.5
**Distance**	10	10.0	10.0	0	25.0

Table 7. Stability levels for results of **New Brunswick** for weighted scenario, with levels for all ranking to remain the same, and levels for half of the rankings to remain the same.

Criteria	Weight	**Stability intervals for rankings to remain the same**		**Stability intervals for 50% of rankings to remain the same**	
		Min weight	Max weight	Min weight	Max weight
**Risk**	40	40.0	40.0	33.3	52.5
**Environment**	25	25.0	25.0	23.9	35.3
**Population**	25	24.2	25.0	13.7	26.1
**Distance**	10	9.6	10.0	0	12.6

Table 8. Stability levels for results of **Nova Scotia** for weighted scenario, with levels for all ranking to remain the same, and levels for half of the rankings to remain the same.

Criteria	Weight	**Stability intervals for rankings to remain the same**		**Stability intervals for 50% of rankings to remain the same**	
		Min weight	Max weight	Min weight	Max weight
**Risk**	40	39.5	53.9	39.5	54.7
**Environment**	25	21.1	32.8	21.1	51.6
**Population**	25	16.7	25.5	13.8	25.5
**Distance**	10	7.0	10.7	0.9	10.7

Table 9. Stability levels for results of **Prince Edward Island** for weighted scenario, with levels for all ranking to remain the same, and levels for half of the rankings to remain the same.

Criteria	Weight	**Stability intervals* for rankings to remain the same**		**Stability intervals for 50% of rankings to remain the same**	
		Min weight	Max weight	Min weight	Max weight
**Risk**	40	29.4	42.9	29.4	46.8
**Environment**	25	17.8	34.8	17.8	34.8
**Population**	25	18.9	44.4	0	44.4
**Distance**	10	7.7	21.7	3.1	21.7

Table 10. Stability levels for results of **Newfoundland and Labrador** for weighted scenario, with levels for all ranking to remain the same, and levels for half of the rankings to remain the same.

Criteria	Weight	**Stability intervals for rankings to remain the same**		**Stability intervals for 50% of rankings to remain the same**	
		Min weight	Max weight	Min weight	Max weight
**Risk**	40	33.3	47.8	33.3	47.8
**Environment**	25	25.0	25.0	10	25.8
**Population**	25	25.0	25.0	21.1	30.2
**Distance**	10	10.0	10.0	9.5	13.7
